# Ultra‐Low Loss Weakly Coupled Few‐Mode Hollow‐Core Anti‐Resonant Fiber Enabling Eight Channels via Differential Wall Thickness and Truncated Tubes

**DOI:** 10.1002/nap2.70177

**Published:** 2026-07-02

**Authors:** Biao Wang, Qiang Shi, Xin Wang, Shuqin Lou, Zhenggang Lian

**Affiliations:** ^1^ Key Laboratory of Communication and Information Systems Beijing Municipal Commission of Education School of Electronic and Information Engineering Beijing Jiaotong University Beijing China; ^2^ Yangtze Optics Electronics Co. Ltd. Wuhan China

**Keywords:** anti‐resonant fiber, few‐mode fiber, microstructured fiber, mode‐division multiplexing, optical fiber design

## Abstract

We propose and analyze a novel, ultra‐low loss, weakly coupled few‐mode hollow‐core anti‐resonant fiber that supports eight spatial modes as independent channels. The design features an optimized, simplified cladding structure with differentiated tube thicknesses. This configuration effectively suppresses coupling between the guided core modes and cladding modes, significantly reducing confinement loss (CL), and mitigating loss fluctuations caused by nesting nodes, while also minimizing surface scattering loss (SSL). Simulation results demonstrate that within the wavelength range of 1460–1680 nm, the effective refractive index difference between adjacent core modes consistently exceeds 1.0 × 10^−4^, ensuring weak mode coupling. The intrinsic loss (CL + SSL) for all eight modes remains below 1.27 × 10^−4^ dB/m across this band, reaching a minimum loss of less than 9.92 × 10^−5^ dB/m at 1550 nm. The loss ratio between higher‐order modes and the fundamental mode is maintained below 15.38 in the operating band. Furthermore, with a bending radius of just 5 cm, the loss of all eight modes does not exceed 1.35 × 10^−4^ dB/m. This work employs a simplified fiber structure that may be easier to manufacture and represents a significant step forward toward practical, low‐loss, high‐capacity, low‐latency mode‐division multiplexing systems.

## Graphical abstract

A novel ultra‐low loss, weakly coupled few‐mode hollow‐core anti‐resonant fiber is realized through truncated tubes and differential wall thickness, which can support eight spatial modes as independent channels. The effective refractive index difference between adjacent modes is greater than 1.0 × 10^−4^, and the intrinsic loss of all target modes at 1550 nm is less than 9.92 × 10^−5^ dB/m.

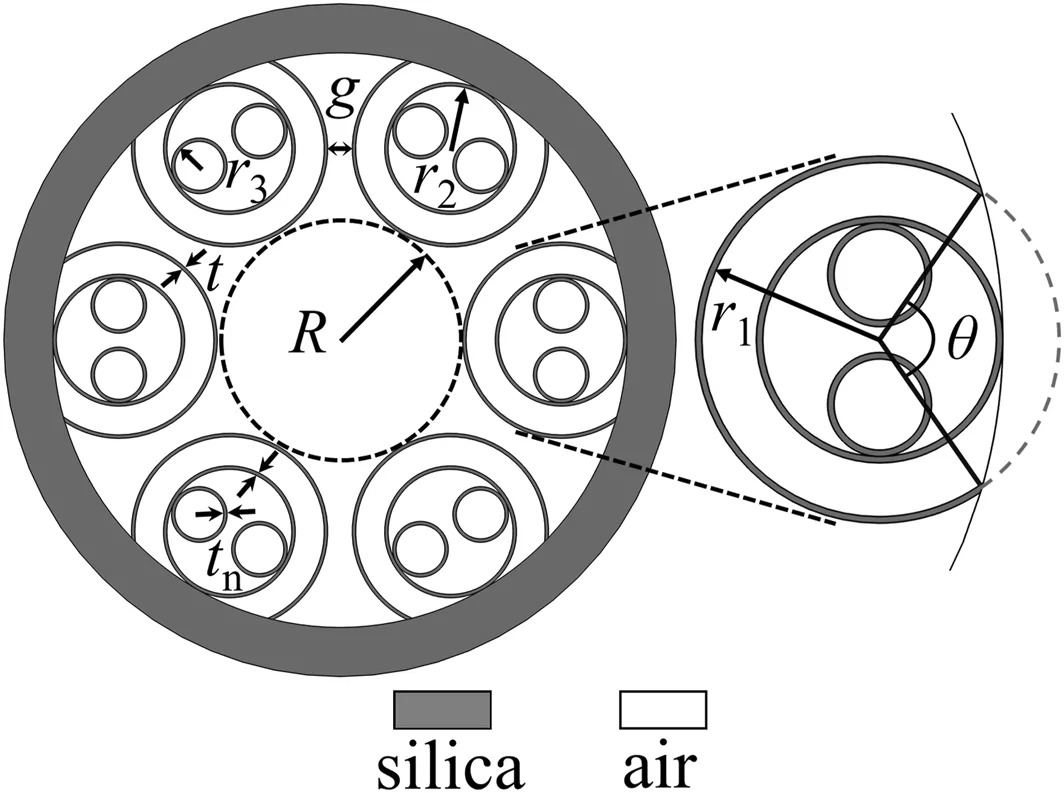

## 1 Introduction

The exponential growth of data traffic driven by the rapid expansion of the Internet and cloud computing is pushing the capacity limit of communication systems based on traditional single‐mode optical fibers (SMFs). Space‐division multiplexing (SDM), particularly mode‐division multiplexing (MDM) using few‐mode fibers (FMFs), has emerged as a pivotal technology to scale the per‐fiber capacity by exploiting orthogonal spatial modes as independent data channels [1, 2, 3]. However, traditional solid‐core FMFs may ultimately fail to meet the demands of high‐speed, low‐latency applications due to the inherent limitations of the substrate materials.

Hollow‐core anti‐resonant fibers (HCARFs), which guide light within an air core, offer a revolutionary platform to overcome these limitations. They exhibit low nonlinearity, ultra‐low loss, reduced delay, ultra‐low chromatic dispersion and high damage threshold [4, 5, 6]. HCARFs possess inherent multimode characteristics [7, 8, 9] due to its unique light‐guiding mechanism through anti‐resonance and inhibited coupling effects [10, 11]. Further customizing the number of modes to support a few‐mode configuration could make it a promising MDM candidate. In FMFs, the mode coupling can be directly reduced by increasing the effective refractive index difference (Δ*n*_eff_) between guiding modes. Research indicates that when the Δ*n*_eff_ between modes exceeds 10^−4^, weak coupling between modes is achieved, effectively suppressing inter‐mode crosstalk [12, 13].

The MDM link based on weakly coupled FMFs can eliminate the need for complex and power‐consuming multiple‐input multiple‐output digital signal processing (MIMO‐DSP) equipment, significantly reducing the power consumption and cost of MDM transmission systems [1, 2]. The combination of MDM technology with weakly coupled few‐mode HCARF (FM‐HCARF) has the potential to exponentially increase the single‐fiber capacity of MDM transmission systems based on traditional solid‐core FMFs while significantly reducing data transmission delays. Additionally, an even greater leap in channel capacity can be achieved in comparison to the current single‐mode HCARF. Consequently, the weakly coupled FM‐HCARF could be a promising candidate for future high‐capacity, energy‐efficient optical networks.

The key challenge in designing FM‐HCARFs for weakly coupled MDM systems lies in maintaining low loss, weak inter‐mode coupling, and wide bandwidth across multiple spatial modes while minimizing loss differences between modes. Recent advances in HCARF design have progressed from supporting two weakly coupled modes [14] to more complex structures supporting five [15] or even eight modes [16]. However, these designs often involve intricate multi‐layer nested claddings, which can compromise bending performance and increase fabrication complexity. For instance, an earlier eight‐mode design required a bending radius exceeding 20 cm to maintain low loss differences [16], limiting practical deployment. Furthermore, prior studies have primarily focused on minimizing confinement loss (CL) and bending loss (BL) [14, 15, 16]. In the low‐loss window, the total intrinsic loss of HCARFs also includes surface scattering loss (SSL) arising from interface roughness [17]. Even for FM‐HCARFs with larger cores, where higher‐order modes may exhibit stronger field intensity at the silica‐air interfaces, SSL could become a significant loss source and must be co‐optimized with CL.

In this paper, we address these challenges by proposing a novel low‐loss, weakly coupled FM‐HCF that supports eight spatial modes (LP_01_–LP_31a_, LP_31b_–LP_41_) as independent channels. By comprehensively evaluating the CL and SSL of the fiber, a more accurate and realistic reflection of the final loss performance of optical fibers can be provided. On the one hand, we suppressed the coupling between the guided core modes and the cladding modes effectively using truncated tubes and a novel nested configuration, thereby reducing CL. On the other hand, we introduced a design with differentiated wall thicknesses in the simplified nested structure to minimize SSL and mitigating loss fluctuations caused by nesting nodes. Through meticulous parameter optimization, the proposed fiber exhibits an inter‐mode Δ*n*_eff_ greater than 1.0 × 10^−4^ across a wide wavelength range from 1460 to 1680 nm, ensuring weak inter‐mode coupling. Remarkably, the intrinsic loss (CL + SSL) for all eight modes remains below 1.27 × 10^−4^ dB/m across this band with a minimum loss below 9.92 × 10^−5^ dB/m at 1550 nm. The loss ratio between higher‐order modes and the fundamental mode is kept below 15.38 within the operating band. Crucially, it delivers exceptional bending performance: for a bending radius of 5 cm, the loss of all eight modes remains low and virtually unaffected, with losses all below 1.35 × 10^−4^ dB/m at 1550 nm. Compared to the previous reports, this new design outperforms previous complex designs in terms of practical feasibility and overall performance.

## 2 Fiber Structure and Numerical Method

The cross‐section of the proposed FM‐HCARF is illustrated in Figure 1. It features a simplified cladding design comprising six identical units surrounding a central air core with radius *R*. Each unit consists of three layers of silica tubes: an outer truncated tube (radius *r*_1_, truncated angle *θ*, and tube wall thickness *t*), an intermediate nested tube (radius of *r*_2_ and tube wall thickness *t*_n_), and an inner nested structure formed by two small circular tubes (radius of *r*_3_ and tube wall thickness *t*_n_) symmetrically arranged and tangent to the intermediate nested tube. And the gap between adjacent cladding units is denoted by *g*. The geometric relationship between these structure parameters can be expressed as

(1) $$r_{1}=\frac{g/2+t-\left(R+t\right)\sin\left(\pi /N\right)}{\sin\left(\pi /N\right)-1}$$

where *N* (*N* = 6) represents the number of cladding units.

**FIGURE 1:**
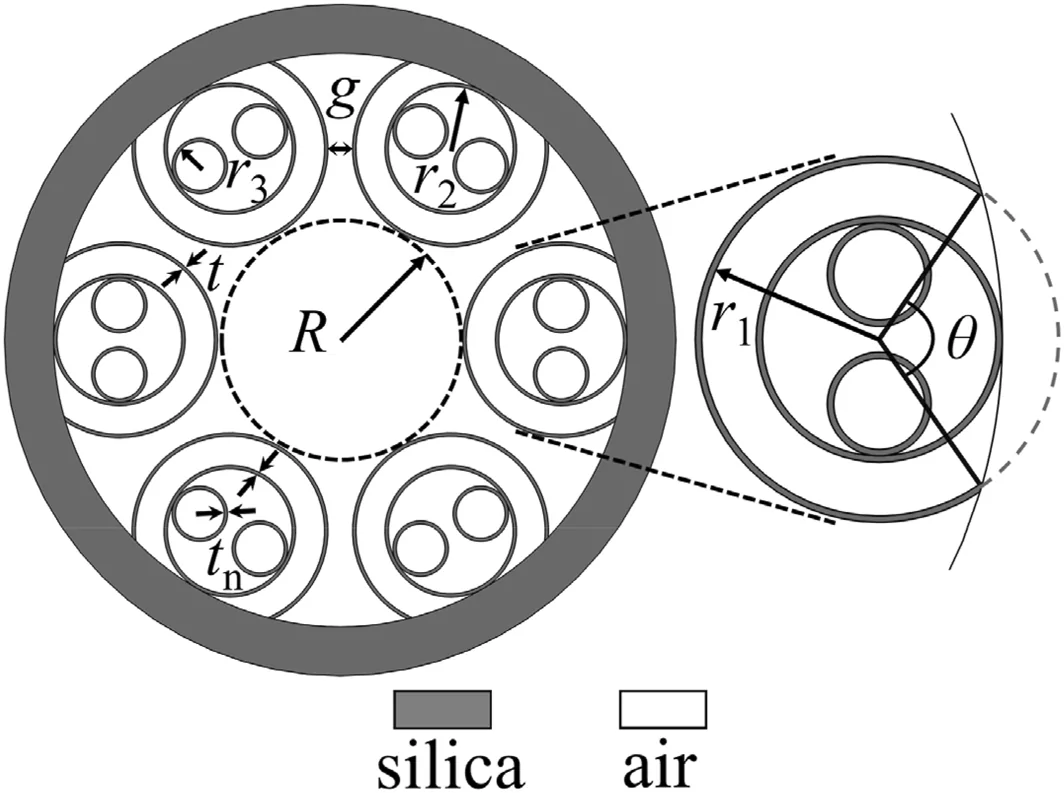
The cross‐section of the proposed fiber and magnified cladding unit.

The finite element method with a perfectly matched layer (PML) was employed for mode analysis and loss calculation using COMSOL Multiphysics software. A PML with a thickness of 12 μm was placed outside the fiber cladding to calculate the mode CL. The grid sizes of the air region and the silica region (including the PML) were set to λ/4 and λ/6, respectively [18]. The CL was calculated by the following formula [19]:

(2) $$\text{CL}=\frac{20}{\ln\;10}\frac{2\pi }{\lambda }\text{Im}\left(n_{\text{eff}\;}\right)$$

where λ is the wavelength, and Im (*n*_eff_) is the imaginary part of the effective refractive index of the mode.

In the simulation, the refractive index of air was set to 1, whereas the refractive index of silica was determined via the Sellmeier equation [20]. For the initial parameters, *t*_cl_, denoting *t* and *t*_n_, was set to the same value of 0.52 μm. Additionally, the other key parameters were configured as follows: *R* = 23 μm, *g* = 2.8 μm, *r*_2_/*r*_1_ = 0.66, *r*_3_/*r*_2_ = 0.4, and *θ* = 100°.

## 3 Optimization of Structural Parameters

### 3.1 Effects of Core Radius and Cladding Gap

The core radius *R* and gap *g* between cladding units are critical parameters that significantly influence the CL of the guiding modes and the effective refractive index difference (Δ*n*_eff_) between adjacent modes. As demonstrated in Figure 2a, increasing *R* from 15 to 27 μm can significantly reduce the CL for all eight modes (LP_01_ to LP_41_). However, an increase in *R* results in a simultaneous decrease in Δ*n*_eff_, as illustrated in Figure 2b. The Δ*n*_eff_ between LP_31b_ and LP_12_ modes is minimal. When *R* exceeds 24 μm, its Δ*n*_eff_ becomes less than 1.0 × 10^−4^. To satisfy the weak coupling condition across the target band, *R* = 23 μm was selected as the optimal value, ensuring the Δ*n*_eff_ between any adjacent modes greater than 1.0 × 10^−4^ and the CL below 2.5 × 10^−5^ dB/m for all eight modes.

**FIGURE 2:**
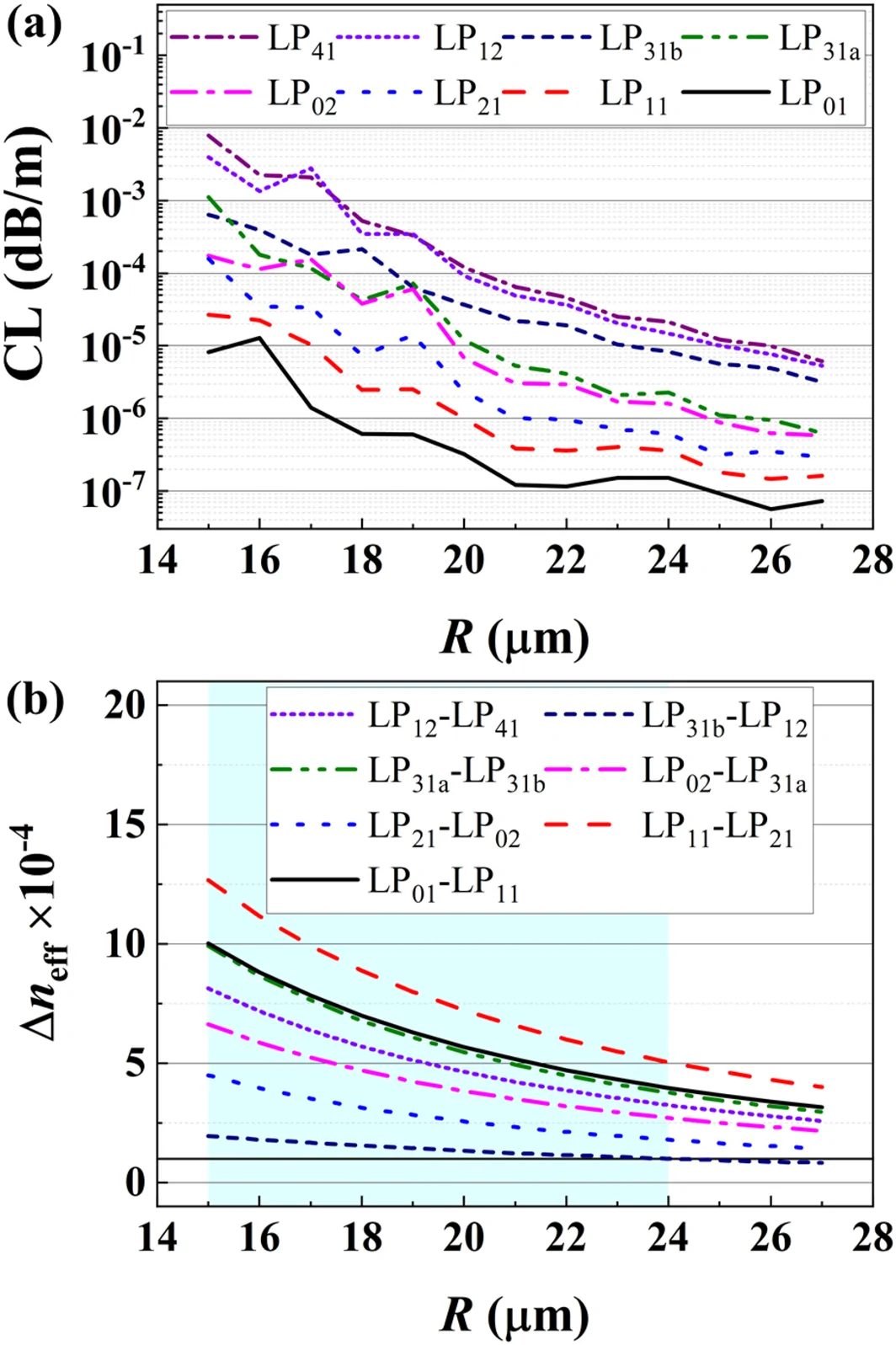
Effects of the core radius on (a) CL of eight guided core modes and (b) Δ*n*_eff_ between adjacent modes of the fiber for *g* = 2.80 μm, *r*_2_/*r*_1_ = 0.66, *r*_3_/*r*_2_ = 0.40, *θ* = 100°, and *t*_cl_ = 0.52 μm at 1550 nm.

Given that the fiber core radius is constant at *R* = 23 μm, the change of gap *g* influences both loss and bandwidth. As demonstrated in Figure 3a, varying *g* from 1 to 5 μm affects CL differently for lower‐order (LP_01_–LP_31a_) and higher‐order (LP_31b_–LP_41_) modes. The lower‐order modes LP_01_–LP_31a_ gradually increase with increasing *g*, whereas the higher‐order modes LP_31b_–LP_41_ exhibit a trend of first decreasing and then increasing. This can be explained that a smaller *g* value corresponds to a larger air area in the cladding unit, which makes LP_31b_–LP_41_ modes in the fiber core more susceptible to coupling with cladding modes and thereby increasing the CL for these three modes. When *g* is large, the increase in CL for LP_01_–LP_41_ modes arises from similar mechanisms: power leakage occurs along the gaps between cladding units, accompanied by mode coupling between the core and cladding units, as illustrated by the contour diagrams of the normalized transverse electric field for the LP_41_ mode in the inset of Figure 3a. In addition to the CL, the gap between the cladding units has a significant impact on the *n*_eff_ of the core modes. As demonstrated in Figure 3b, the spectral characteristics of the minimum Δ*n*_eff_ are depicted as a function of the gap *g*. It is evident that when *g* is set to 2, 2.5, and 3 μm, the respective minimum wavelengths that satisfy the weak coupling condition are 1440, 1470, and 1520 nm, respectively. It has been demonstrated that as *g* increases, the weak coupling transmission bandwidth of the fiber decreases. Therefore, it can be concluded that if *g* is set to 2.5 μm, this will result in CL below 2.5 × 10^−5^ dB/m for all eight modes while maintaining a broader weak coupling transmission spectrum.

**FIGURE 3:**
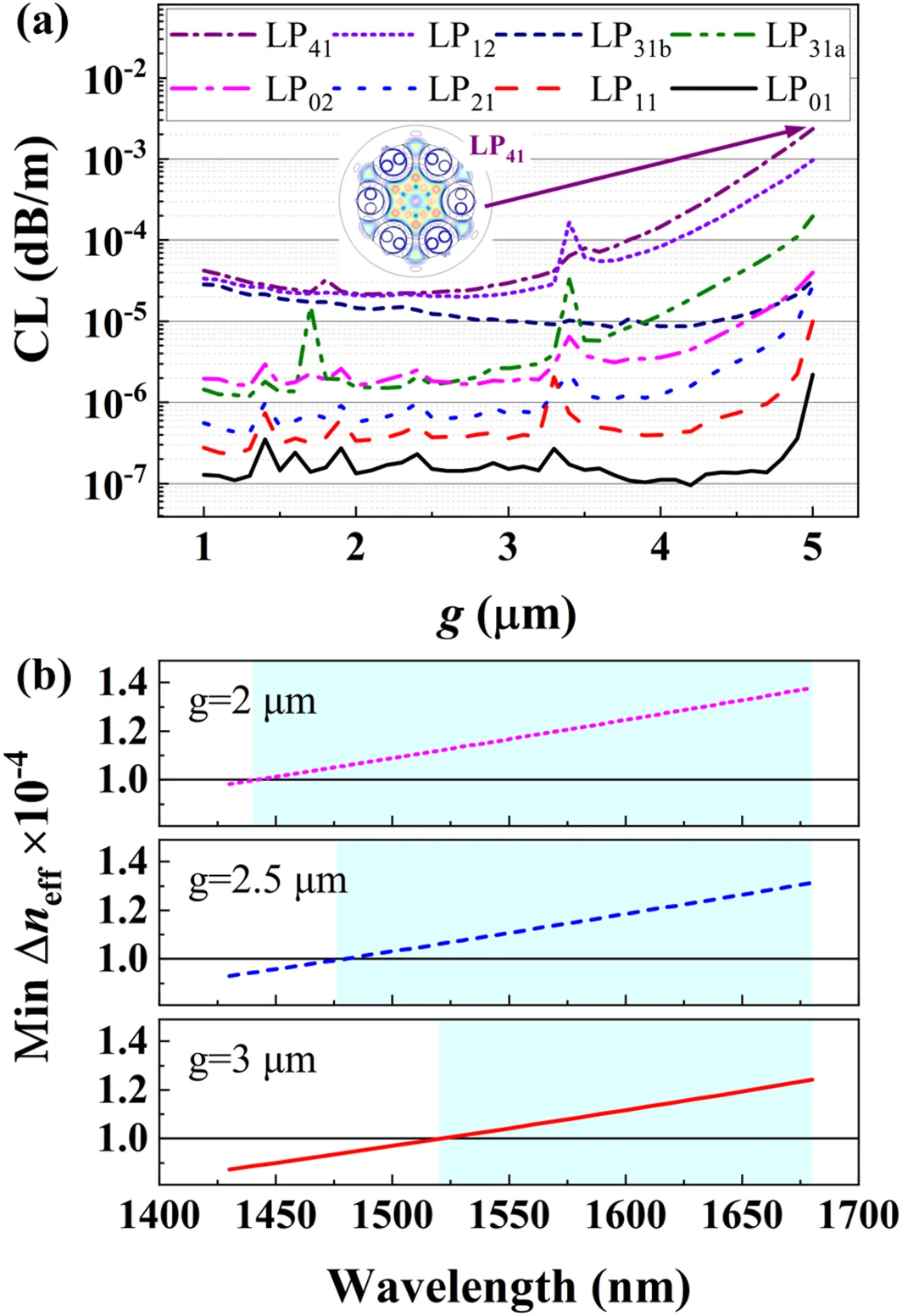
Effects of the gap *g* between adjacent cladding units on (a) CL of eight guided core modes at 1550 nm and (b) minimum Δ*n*_eff_ between adjacent modes in the wavelength range between 1430 and 1680 nm for *R* = 23 μm, *g* = 2.80 μm, *r*_2_/*r*_1_ = 0.66, *r*_3_/*r*_2_ = 0.40, *θ* = 100°, and *t*_cl_ = 0.52 μm. The inset in (a) depicts the contour diagram of the normalized transverse electric field for LP_41_ mode.

### 3.2 Effects of Nested Tubes

The ratios *r*_2_/*r*_1_ and *r*_3_/*r*_2_, along with the truncation angle *θ* of the outer tube, were optimized to minimize the CL for all eight modes. As illustrated in Figure 4, the maximum CL for all eight guided modes is influenced by *r*_2_/*r*_1_ and *θ*. For a given value of *θ*, an insufficiently large *r*_2_/*r*_1_ ratio serves to diminish the confinement effect exerted by the middle and inner tubes on the mode. For instance, at *θ* = 110°, as demonstrated in Figure 4 inset, at *r*_2_/*r*_1_ = 0.54, the intermediate tube and inner tube are far from the guided mode, causing the field to penetrate the outer tube and leak through the gap between the outer and middle tubes. Conversely, an excessively large *r*_2_/*r*_1_ ratio causes coupling between the guided mode and the cladding mode. Again for *θ* = 110°, as shown in Figure 4 inset, at *r*_2_/*r*_1_ = 0.74, coupling between the core mode and the cladding mode between the intermediate and inner tubes results in increased CL. The maximum CL exhibits similar trends across different *θ* values as *r*_2_/*r*_1_ varies. However, excessively large or small *θ* values weaken the cladding's confinement effect on the guided mode. The curve for *θ* = 110° in Figure 4 exhibits the smallest maximum CL of 1.46 × 10^−5^ dB/m at *r*_2_/*r*_1_ = 0.65. Therefore, it can be concluded that the minimization of CL for all eight guided modes is achieved by setting *r*_2_/*r*_1_ = 0.65 and *θ* = 110°.

**FIGURE 4:**
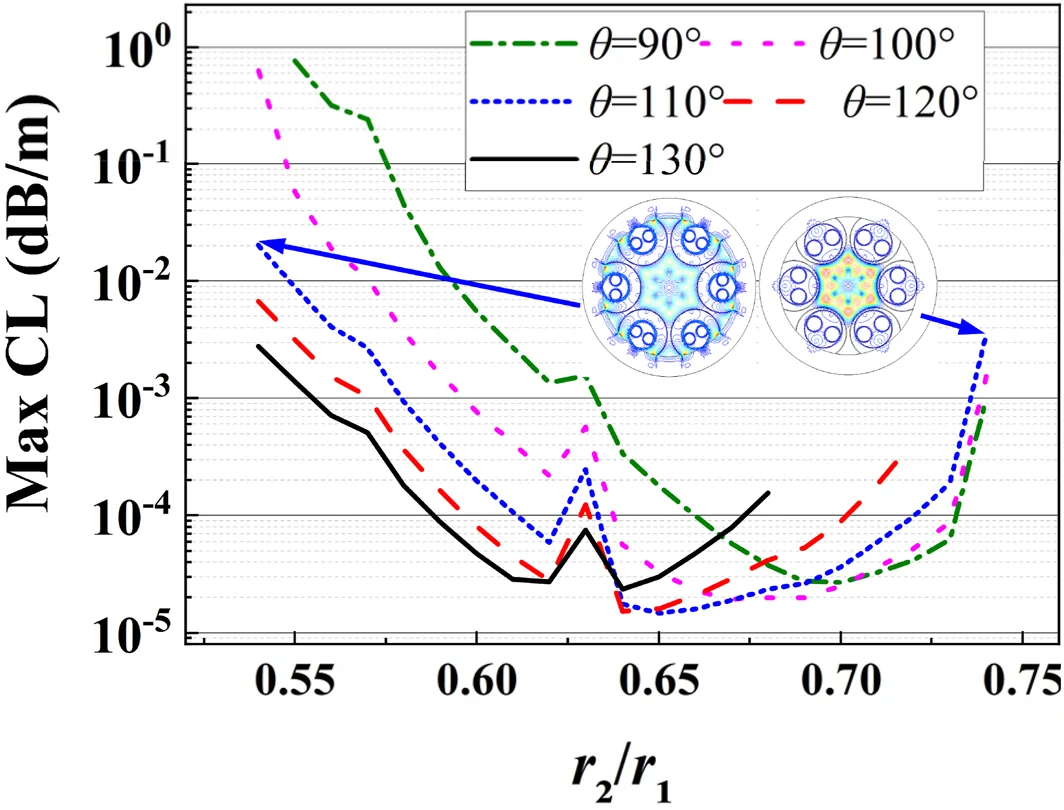
Effects of the truncation angle of the outer tube and the intermediate tube radius on maximum CL in the LP_01_‒LP_41_ modes of the fiber for *R* = 23 μm, *g* = 2.5 μm, *r*_3_/*r*_2_ = 0.40, and *t*_cl_ = 0.52 μm at 1550 nm. The inset depicts the contour diagrams of the normalized transverse electric field for the guided modes.

Then, we turn to observe the effect of *r*_3_/*r*_2_, as demonstrated in Figure 5. It can be seen that the CL of all eight modes exhibits a substantial increase as *r*_3_/*r*_2_ decreases. This phenomenon can be attributed to the fact that decreasing *r*_3_/*r*_2_ results in an augmentation of the separation between the inner tubes, consequently precipitating the leakage of the guided mode's optical power. Moreover, the higher‐order modes are more susceptible to coupling with the cladding modes, as demonstrated in the inset of Figure 5. When *r*_3_/*r*_2_ decreases to 0.32, the LP_41_ mode couples with the cladding mode between the middle tube and inner tube, resulting in a significant increase in CL. It should be noted that an excessively large *r*_3_/*r*_2_ ratio can result in the two inner tubes making contact, thereby causing coupling between the core mode and the tube wall mode. This, in turn, leads to an increase in CL. Therefore, selecting *r*_3_/*r*_2_ = 0.4 ensures all eight modes with relatively low CL.

**FIGURE 5:**
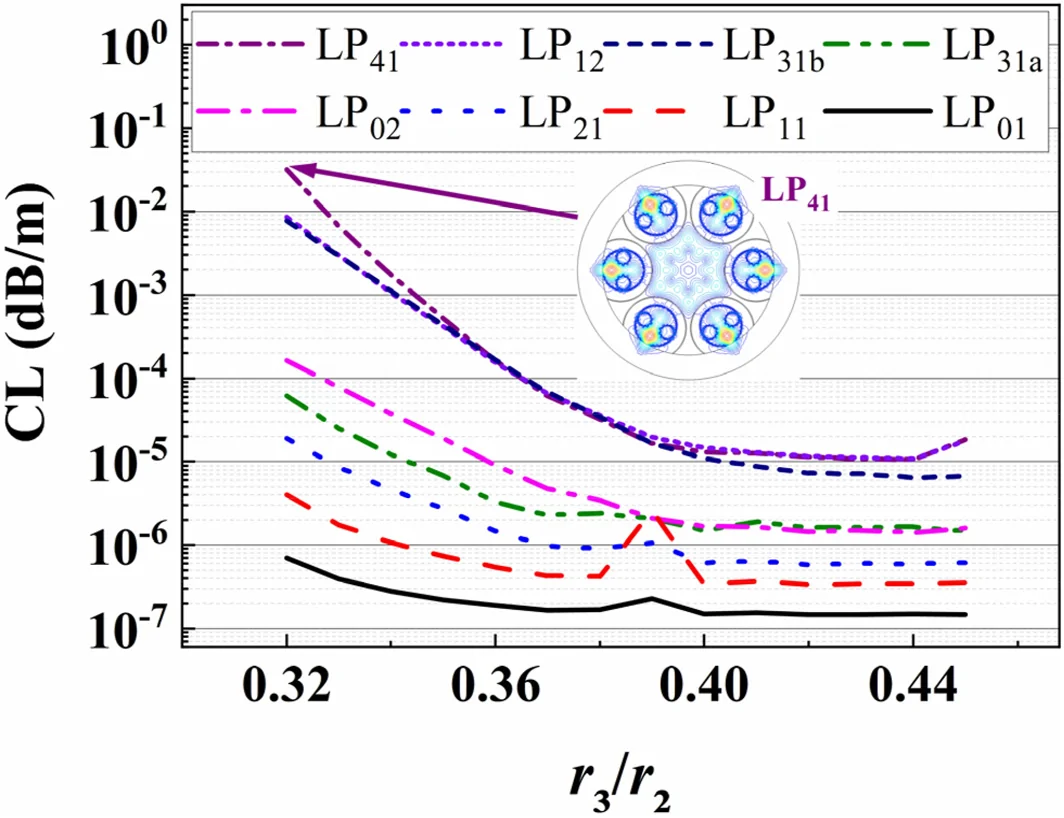
Effects of the intermediate tube radius on CL of eight guided core modes of the fiber for *R* = 23 μm, *g* = 2.5 μm, *r*_2_/*r*_1_ = 0.65, *θ* = 110°, and *t*_cl_ = 0.52 μm at 1550 nm. The inset depicts the contour diagrams of the normalized transverse electric field for LP_41_ mode.

In other words, the CL in HC‐ARFs is primarily governed by the coupling between the core‐guided modes and the lossy modes of the glass cladding. The nested tubes (with diameters *r*_2_ and *r*_3_) act as additional anti‐resonant layers. By adjusting their sizes, we can tune the effective index of the cladding modes. Specifically, when a particular *r*_2_ or *r*_3_ value causes the effective index of a cladding mode to closely match that of a core mode (phase‐matching), strong resonant coupling occurs, leading to a sharp increase in CL for that core mode. By optimizing *r*_2_ and *r*_3_, we can ensure that the effective index of the cladding modes is sufficiently different (phase‐mismatched) from all target core modes. This effectively suppresses the resonant coupling, resulting in low loss for the desired modes.

### 3.3 Effects of Wall Thickness

Optimizing the geometric parameters of the cladding tube suppresses the coupling between the LP_01_–LP_41_ modes and the cladding modes. This section analyzed the influence of the cladding tube wall thickness (*t* and *t*_n_) on the SSL of eight guided modes and further optimized the CLs. The SSL of the guided modes can be calculated as follows [9, 17, 21]:

(3) $$\text{SSL}\left(\text{dB}/\text{km}\right)=F\times \eta \times {\left(\lambda /\lambda_{0}\right)}^{-3}$$

where *F* represents normalized interface electric field intensity, *η* is the calibration factor, and λ_0_ = 1550 nm. Figure 6 shows the SSL of eight core modes as a function of cladding wall thickness *t*_cl_. It can be found that higher‐order modes exhibit higher SSL at the same *t*_cl_. This stems from the stronger electric field intensity of higher‐order modes at the silica‐air interface, resulting in an increase in SSL. Simultaneously, within the range of *t*_cl_ from 0.3 to 0.6 μm, the SSL of all eight modes first decreases and then increases and attains a minimum around *t*_cl_ = 0.4 μm.

**FIGURE 6:**
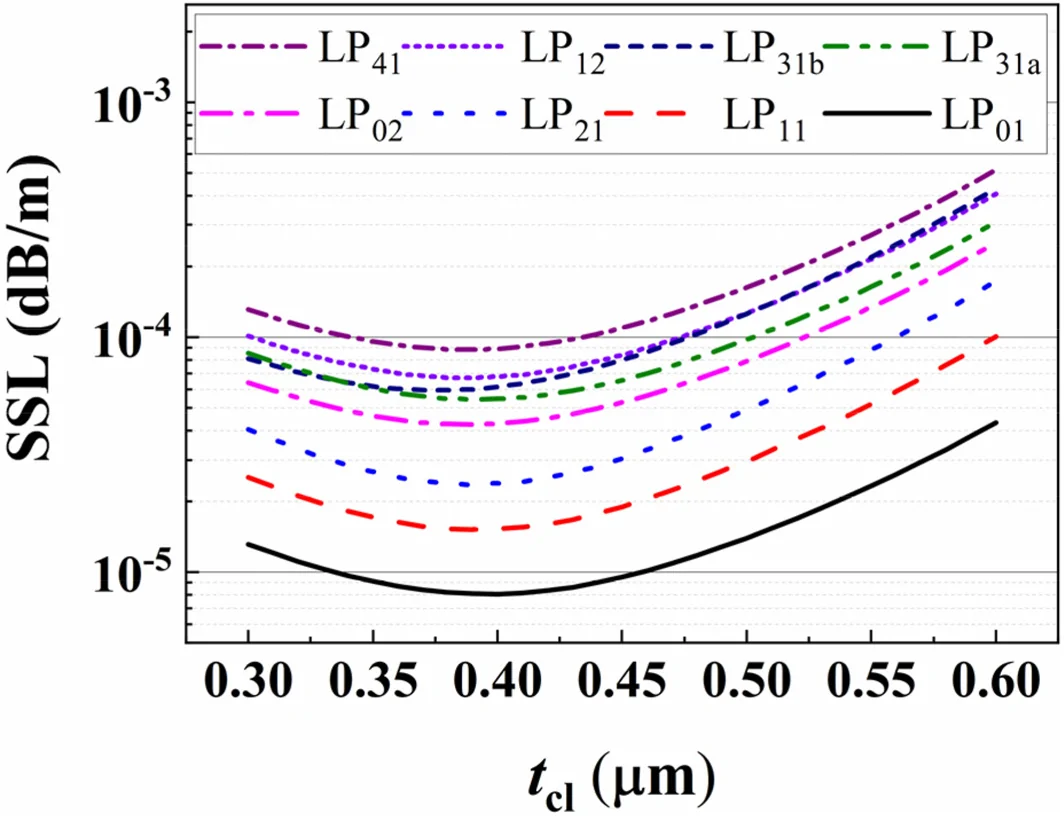
Effects of the wall thickness of cladding tubes on SSL of the LP_01_–LP_41_ modes of the fiber for *R* = 23 μm, *g* = 2.50 μm, *r*_2_/*r*_1_ = 0.65, *r*_3_/*r*_2_ = 0.40, and *θ* = 110° at 1550 nm.

Especially, the effect of wall thickness on SSL, CL, and total intrinsic loss of the highest order mode LP_41_ is shown in Figure 7. For a small *t*_cl_ of 0.36 μm, the average CL is higher than the SSL; therefore, CL dominates the intrinsic loss. However, as *t*_cl_ increases beyond 0.48 μm, SSL surpasses CL and becomes the dominant factor in intrinsic loss. When *t*_cl_ increases to 0.52 μm, the SSL of the LP_41_ mode at 1550 nm rises from a minimum value of 8.93 × 10^−5^ dB/m at *t*_cl_ = 0.4 μm to 1.44 × 10^−4^ dB/m. Further increasing *t*_cl_ to 0.54 μm causes a significant increase in SSL to 2.43 × 10^−4^ dB/m. Notably, at small *t*_cl_ values, the loss spectrum of the LP_41_ mode displays considerable fluctuations. These fluctuations may originate from Fano resonance at certain wavelengths caused by the nested‐tube connection points [22]. It has been demonstrated that *t*_cl_ values greater than 0.48 μm have a significant mitigating effect on these fluctuations. To further achieve a flatter loss spectrum and low‐loss transmission capability, the outer tube wall thickness *t* was set to 0.4 μm, whereas the intermediate and inner tube wall thicknesses *t*_n_ were set to 0.52 μm, thereby differential wall thickness design (*t* = 0.4 μm, *t*_n_ = 0.52 μm) was adopted.

**FIGURE 7:**
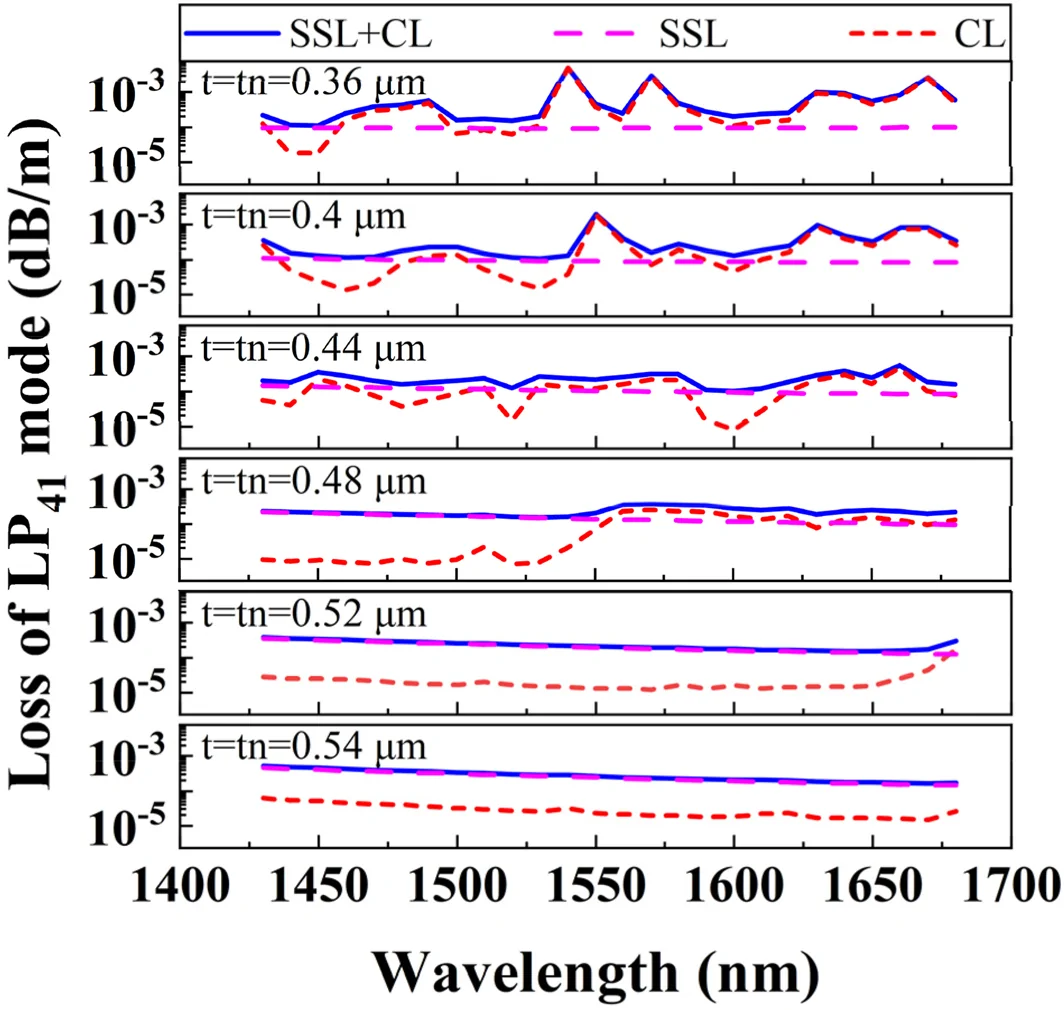
Effects of the wall thickness of cladding tubes on SSL, CL, and total loss of the LP_41_ mode in the wavelength range between 1430 and 1680 nm for the fiber with *R* = 23 μm, *g* = 2.50 μm, *r*_2_/*r*_1_ = 0.65, *r*_3_/*r*_2_ = 0.40, and *θ* = 110°.

The final optimized parameters are summarized in Table 1.

**TABLE 1:** Parameters of the optimized fiber design.

Parameter	Value
*g*	2.50 μm
*R*	23 μm
*r*_2_∕*r*_1_	0.65
*r*_3_∕*r*_2_	0.40
*θ*	110°
*t*	0.4 μm
*t*_*n*_	0.52 μm

## 4 Fiber Performance

### 4.1 Loss and Bandwidth

Figure 8 presents the comprehensive performance of the optimized fiber over the 1430–1680 nm range. It can be observed from Figure 8c that the Δ*n*_eff_ between all adjacent mode pairs consistently exceeds 1 × 10^−4^ within the wavelength range from 1460 to 1680 nm, ensuring weak coupling and minimal inter‐channel crosstalk for MDM. As shown in Figure 8a, the total intrinsic loss of all eight weakly coupled modes (including CL and SSL) remains below 1.27 × 10^−4^ dB/m across the 1460–1680 nm band, with a minimum of 9.92 × 10^−5^ dB/m at 1550 nm. Additionally, eight weakly coupled modes exhibit ultra‐low loss differences, as shown in Figure 8b. The loss ratio (LR) between the higher‐order modes and the fundamental mode is consistently below 15.38 to minimize damage caused by mode dependent loss in MDM fiber links [23, 24]. Verification of the mode separation characteristics was conducted through the utilization of differential group delay (DGD) between adjacent modes. As demonstrated in Figure 8d, the DGD between adjacent modes in the waveband of 1460–1680 nm exhibits a minimum value of 0.38 ps/m at 1460 nm, which is significantly greater than 0.1 ps/m. This value increases with the increasing wavelength, thereby demonstrating that these guided modes can be well separated [25]. We also calculated the effective mode field areas (A_eff_) of eight modes, all of which are greater than 750 μm^2^ in the weak coupling wavelength range, as shown in Figure 9. This further demonstrates the ultra‐low nonlinearity characteristic of our proposed fiber.

**FIGURE 8:**
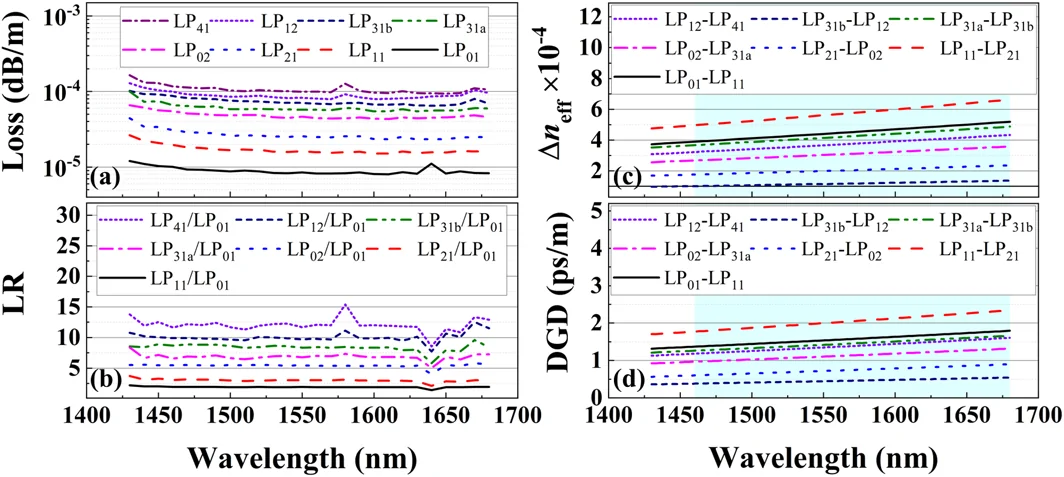
(a) Total loss and (b) LR of the LP_01_–LP_41_ modes; (c) Δ*n*_eff_ and (d) DGD between adjacent modes in the wavelength range between 1430 and 1680 nm for the fiber with *R* = 23 μm, *g* = 2.50 μm, *r*_2_/*r*_1_ = 0.65, *r*_3_/*r*_2_ = 0.40, *θ* = 110°, *t* = 0.4 μm, and *t*_n_ = 0.52 μm.

**FIGURE 9:**
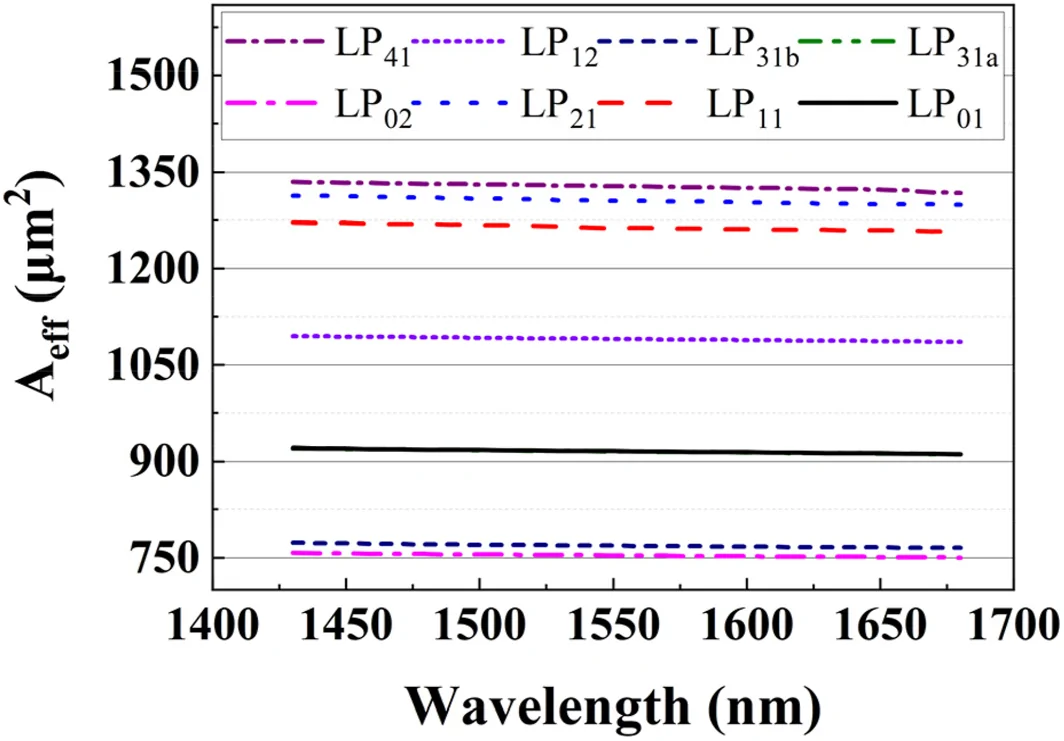
Effective mode field area of the LP_01_–LP_41_ modes in the wavelength range between 1430 and 1680 nm for the fiber with *R* = 23 μm, *g* = 2.50 μm, *r*_2_/*r*_1_ = 0.65, *r*_3_/*r*_2_ = 0.40, *θ* = 110°, *t* = 0.4 μm, and *t*_n_ = 0.52 μm.

### 4.2 Bending Performance

Bending performance is crucial for practical deployment. Bending primarily affects the transmission loss of guided modes, which can be analyzed by using conformal mapping techniques [26]. For the fiber cross‐section illustrated in the inset of Figure 10a, bending in the *x*‐direction is more likely to cause core‐cladding mode coupling than that in the *y*‐direction [14, 27]. As shown in Figure 9, for the bending radius exceeding 5 cm, the loss of the eight modes is not significantly affected by bending and the loss for all eight guided modes remains below 1.35 × 10^−4^ dB/m at 1550 nm. If the bending radius is further reduced, higher‐order core modes begin to couple with cladding modes before the lower‐order modes, resulting in increased loss, as shown in the inset of Figure 10a. Notably, even when the bending radius is reduced to 3 cm or smaller, the fiber can still support at least six low‐loss modes (LP_01_–LP_31b_). However, considering more supporting propagation modes, we set a critical bending radius of 5 cm to enable low‐loss guidance of the eight weakly coupled modes. Additionally, the bending loss ratio (BLR) between the higher‐order modes and the fundamental mode was calculated, as shown in Figure 10b. Notably, provided the bending radius exceeds 5 cm, the BLR of the higher‐order modes does not undergo significant changes and remains below 13.1. This represents a significant improvement over the previously reported eight‐mode design which required the bending radius greater than 20 cm [16], highlighting the superior robustness and practicality of our simplified structure.

**FIGURE 10:**
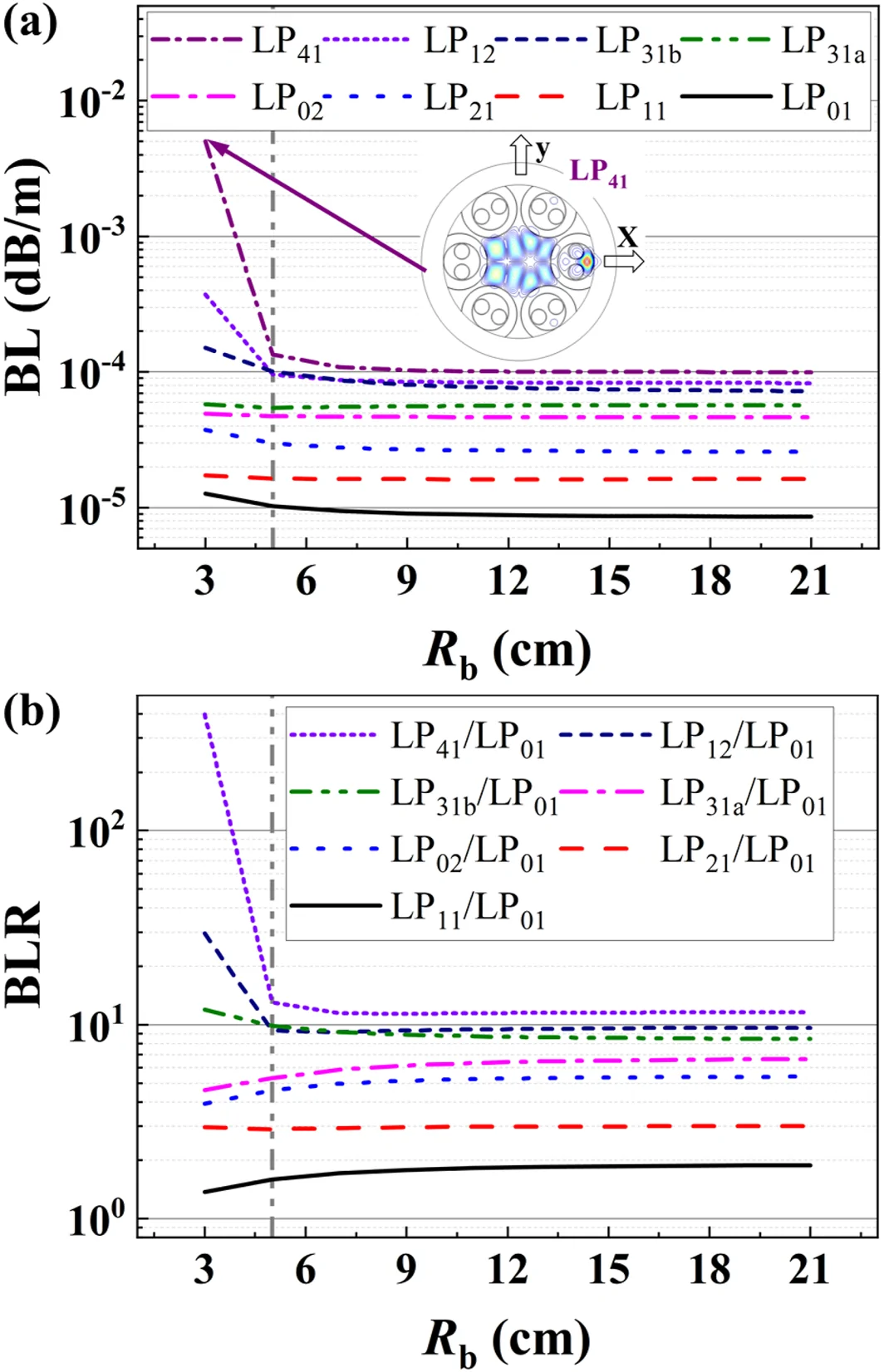
Effects of the bending radius on (a) bending loss and (b) BLR of the LP_01_–LP_41_ modes for the fiber with *R* = 23 μm, *g* = 2.50 μm, *r*_2_/*r*_1_ = 0.65, *r*_3_/*r*_2_ = 0.40, *θ* = 110°, *t* = 0.4 μm, and *t*_n_ = 0.52 μm at 1550 nm. The inset in (a) depicts the normalized transverse electric field contour lines for the guided mode.

### 4.3 Fabrication Tolerance

The proposed HCARF is typically fabricated using the well‐established stack‐and‐draw method [28], which comprises two sequential drawing stages. First, silica capillaries are placed inside a jacket tube of a specific diameter and secured. This brings the outer wall of the capillary tube into contact with the inner wall of the jacket tube, forming a preform. This preform is then drawn into an intermediate cane with a diameter of several millimeters on a drawing tower. In the second stage, this cane is used as a new preform and is drawn into the final fiber. During the fiber drawing process, inert gases are applied separately to the core and cladding capillary regions, enabling precise independent pressure control in each region. This applied pressure counteracts the effects of surface tension and helps to control the final fiber geometry. It is worth noting that, for the optical fiber proposed in this work, the outermost capillary tubes must first be cut at a predetermined angle using a high‐power laser before stacking into the preform [22]. This precise cutting is essential for achieving the desired truncated tube geometry. The adoption of advanced manufacturing techniques has been demonstrated to ensure manufacturing variations within ± 1% of the fiber structure parameters [29]. The fabrication difficulty for the proposed fiber is how to keep the position of two inner tubes in the cladding unit. Here, we analyze the impact of potential manufacturing imperfections, focusing on the offset angle of inner tubes. When the inner tube is offset toward the fiber core, the offset angle is defined as less than zero; conversely, it is defined as greater than zero, as shown in the inset of Figure 11a. Figure 11a shows the effect of the inner tube offset angle on the transmission loss of eight modes under optimized structural parameters. It can be seen that the loss characteristics remain stable for offset angles between −10° and +15°. Beyond this angle range, the loss begins to increase and the loss ratio of the higher modes to the fundamental mode also increases significantly, as depicted in Figure 11b. It can be concluded that the range of offset angles within which fiber performance remains unaffected is between −10° and +15°. In actual manufacturing, the tube offset angle typically falls within ± 5° [30]. This demonstrates that the proposed fiber exhibits high fabrication tolerance for fluctuations in structural parameters during the manufacturing process.

**FIGURE 11:**
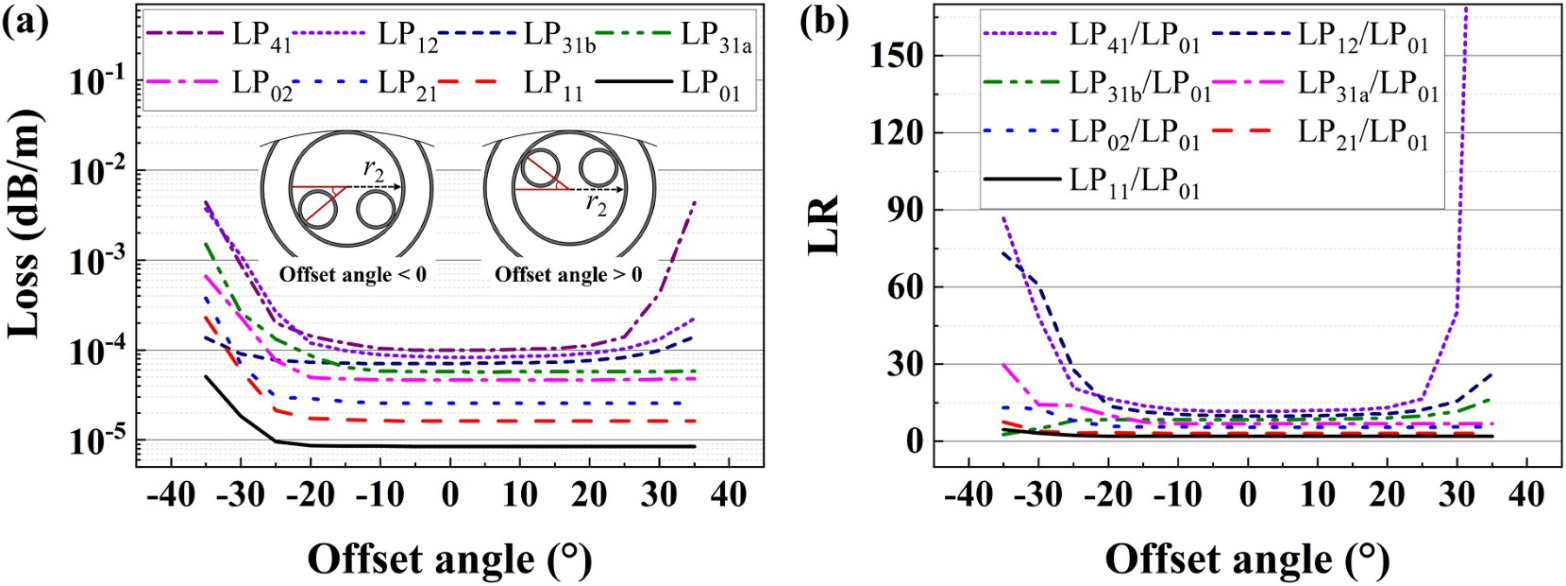
Effects of the inner tube offset angle on (a) loss and (b) LR of the LP_01_–LP_41_ modes of the fiber for *R* = 23 μm, *g* = 2.50 μm, *r*_2_/*r*_1_ = 0.65, *r*_3_/*r*_2_ = 0.40, *θ* = 110°, *t* = 0.4 μm, and *t*_n_ = 0.52 μm at 1550 nm. The inset in (a) depicts the offset angle of the inner nested tube.

## 5 Comparison With Prior Art and Discussion

Compared to existing research on FM‐HCARFs [14, 15, 16], the fiber proposed in this work shows significant improvements in mode count or loss characteristics. In comparison to the eight‐mode fiber described in ref. [15], which uses a complex, three‐layer, nested structure and necessitates a bending radius of over 20 cm, the fiber presented in this study achieves the same mode count and lower loss properties via a considerably more simplified cladding structure. Furthermore, its critical bending radius is reduced to just 5 cm. This advancement enhances the practical feasibility of dense packaging. More attention should be given to the fact that the co‐optimization of CL and SSL also provides a more complete and realistic loss assessment than studies focusing solely on CL. The simplified structure, achieved through strategic parameter optimization rather than increased geometric complexity, suggests potentially easier fabrication compared to highly nested design, without sacrificing performance.

Although the HCARFs can support multiple low‐loss guided core modes, its guidance mechanism introduces significant differential loss [31]. The advanced work reported in refs. [32, 33] increased the number of modes supported by weakly coupled HCARF to four or seven. However, these approaches neglected differences in loss between modes, which could make it difficult to accurately evaluate their performance in MDM. These works may offer significant performance improvements for single‐channel multi‐mode transmission applications [9, 31, 34]. Finally, Table 2 summarizes the performance of representative weakly coupled FM‐HCARF designs.

**TABLE 2:** Summary of the optical properties of different weakly coupled few‐mode hollow‐core anti‐resonant fibers.

References (year)	Mode count	Wavelength range	Max loss (dB/m)	Loss ratio	Bend radius
2020 [14]	2	1530–1565 nm	< 3.1 × 10^−3^ (CL only)	—	10 cm
2022 [15]	5	1330–1630 nm	< 3 × 10^−4^ (CL only)	< 100	6 cm
2024 [16]	8	1480–1670 nm	< 1.72 × 10^−4^ (CL only)	< 100	20 cm
2025 [33]	7	1100–1600 nm	< 1 × 10^−4^ (CL only)	—	7 cm
This work	8	1460–1680 nm	< 1.27 × 10^−4^ (CL + SSL)	< 15.38	5 cm

## 6 Conclusion

We have proposed and numerically validated a novel, simplified low‐loss weakly coupled few‐mode HCARF design that supports eight weakly spatial mode groups (LP_01_, LP_11_, LP_21_, LP_02_, LP_31a_, LP_31b_, LP_12_, and LP_41_) as independent data channels. The use of truncated tubes and a novel nested configuration effectively suppresses coupling between the guide core mode and cladding modes while concomitantly achieving a substantial reduction in CL. The differential wall thickness design minimizes mode SSL while mitigating loss fluctuations caused by nesting nodes. Through co‐optimization of CL and SSL, along with a differentiated tube thickness strategy, the fiber achieves remarkable performance: a minimum intrinsic loss of less than 9.92 × 10^−5^ dB/m for all modes at 1550 nm and the intrinsic loss below 1.27 × 10^−4^ dB/m for all modes over a 220nm bandwidth (1460–1680 nm), loss ratio between higher‐order modes and the fundamental mode below 15.38, inter‐mode effective index differences consistently above 1.0 × 10^−4^, and robust bending performance with a critical radius of just 5 cm. The design maintains high fabrication tolerance. This work demonstrates a significant step toward practical, high‐capacity, and energy‐efficient MDM systems using hollow‐core fiber technology.

## Author Contributions

**Biao Wang:** methodology, visualization, writing – original draft. **Qiang Shi:** investigation. **Xin Wang:** writing – review and editing, supervision, investigation. **Shuqin Lou:** writing – review and editing, supervision, project administration, funding acquisition. **Zhenggang Lian:** writing – review and editing, project administration, funding acquisition, supervision.

## Funding

Supported by the Fundamental Research Funds for the Central Universities (2025YJS019); National Natural Science Foundation of China (12174022).

## Conflicts of Interest

The authors declare no conflicts of interest.

## Data Availability

The data that support the findings of this study are available from the corresponding author upon reasonable request.
